# Do Tumor SURVIVIN and MDM2 Expression Levels Correlate with Treatment Response and Clinical Outcome in Isolated Limb Perfusion for In-Transit Cutaneous Melanoma Metastases?

**DOI:** 10.3390/jpm13121657

**Published:** 2023-11-28

**Authors:** Francesco Russano, Paolo Del Fiore, Fortunato Cassalia, Clara Benna, Luigi Dall’Olmo, Marco Rastrelli, Simone Mocellin

**Affiliations:** 1 Soft-Tissue, Peritoneum and Melanoma Surgical Oncology Unit, Veneto Institute of Oncology IOV-IRCCS, 35128 Padua, Italy; francesco.russano@iov.veneto.it (F.R.); luigi.dallolmo@unipd.it (L.D.);; 2 Unit of Dermatology, Department of Medicine, University of Padua, 35121 Padua, Italy; 3 Department of Surgical, Oncological and Gastroenterological Sciences (DISCOG), University of Padua, 35128 Padua, Italy; clara.benna@unipd.it

**Keywords:** isolated limb perfusion, ILP, in-transit melanoma metastases, SURVIVIN, MDM2

## Abstract

Isolated limb perfusion (ILP) involves the local administration of high doses of anticancer drugs into a limb affected by unresectable locally advanced tumors (with special regard to in-transit melanoma metastases), minimizing systemic side effects. Tumor response to anticancer drugs may depend on the expression of apoptosis-related genes, such as SURVIVIN and MDM2. This retrospective cohort study investigated the association between tumor SURVIVIN and MDM2 expression levels and treatment response or clinical outcomes in patients undergoing ILP for in-transit melanoma metastases. The study cohort consisted of 62 patients with in-transit metastases who underwent ILP with tumor necrosis factor (TNF) and melphalan. Tissue samples were taken from the in-transit metastases, and RNA was extracted for gene expression analysis. Patients’ response to treatment was assessed using clinical and radiological criteria two months after ILP, and disease response was classified as complete, partial, or stable/progressive disease. Disease-free survival (DFS) and overall survival (OS) were also analyzed. Expression of SURVIVIN and/or MDM2 was observed in 48% of patients; in these cases, complete response to ILP occurred in 40% of cases, with the overall response rate (complete + partial) being 85%. Patients with expression of MDM2 alone had a lower complete response rate (28%), while patients with expression of SURVIVIN alone had a higher complete response rate (50%). The combined expression of MDM2 and SURVIVIN resulted in a complete response rate of 30%. Patients without expression (of SURVIVIN or MDM2) had the highest complete response rate (58%). Survival analysis showed that high MDM2 expression was independently associated with a lower probability of a complete response to ILP. In addition, patients with MDM2 expression were three times more likely to have an incomplete response to ILP. This study highlights the importance of considering SURVIVIN and MDM2 expression in patients undergoing ILP for in-transit cutaneous melanoma metastases. High MDM2 expression was found to be an independent factor associated with a reduced likelihood of achieving a complete response to ILP, suggesting potential mechanisms of chemoresistance. These data support further research to explore the role of already available targeted therapies (i.e., MDM2 inhibitors) in improving tumor response to ILP in patients with in-transit melanoma metastases.

## 1. Introduction

It is estimated that in-transit metastases occur in 2–10% of patients with cutaneous melanoma, with the lower limb being the main site in 70% of cases. Surgical excision of the disease is indicated when the lesions are small and few in number, but amputation should be considered when the functionality of the limb is severely impaired or when hygienic conditions are poor. If the extent of the disease contraindicates surgical excision, isolated limb perfusion (ILP) may be used as amputation does not appear to offer any benefit in terms of disease-free survival (DSF) and the efficacy of systemic chemotherapy in local control of the disease is poor. ILP is a well-codified locoregional treatment that allows high doses of cytostatic drugs to be delivered to the affected limb. This technique is quite complex and requires careful monitoring of drug leakage into the systemic circulation and limb temperature to avoid serious and systemic side effects. Melphalan, an alkylating cytotoxic agent, has been used as the reference drug in this procedure since its first clinical application, with a complete response rate of around 40–50% and overall response rate up to 80%. Since the early 1990s, recombinant human tumor necrosis factor (TNF) has been introduced into ILP in combination with melphalan, with a significant increase being observed in the complete response rate, reaching 60–80% in some series, and an overall response rate of around 90% [1,2]. Despite most studies showing a better CR rate using ILP with melphalan plus TNF, some studies have failed to demonstrate a significant difference between the CR rate obtained using ILP with single-agent melphalan and ILP with melphalan plus TNF [3,4]. We hypothesized that this discrepancy may be caused by some mechanism of chemoresistance to TNF. Furthermore, despite the encouraging results obtained with ILP as a treatment for in-transit metastases from limb melanoma, the problem of disease relapse/progression remains unsolved. In fact, this event occurs in at least 60% of patients with a complete response after ILP. We hypothesized that this phenomenon might be related to the same abovementioned variations in expression levels of genes encoding proteins involved in the regulation of the apoptosis machinery.

TNF is a cytokine that has three main effects: antiangiogenic (acting on the endothelial cells of the tumor vasculature), stimulation of the immune response, and direct toxicity mediated by apoptosis. It also facilitates the penetration of melphalan into the tumor [5]. Preclinical studies have shown that p53 is involved in the regulation of the cytotoxic action of TNF-α and that its loss of function can contribute to the resistance of tumor cells to TNF-α [6,7,8]. However, the molecular mechanisms underlying the response to ILP with TNF and melphalan are basically unknown. In this study, we investigated the correlation between the tumor gene expression levels of two key regulators of apoptosis—SURVIVIN and MDM2—and the tumor response or clinical outcomes in patients treated with ILP for in-transit melanoma metastases of the limbs.

### 1.1. SURVIVIN

SURVIVIN is a member of the inhibitor of apoptosis protein (IAP) family that is located on chromosome 17q25 and encodes a 16.5 kDa protein. Cell death is regulated by antiapoptotic proteins, such as members of the Bcl-2 family and inhibitor of apoptosis proteins (IAP) [9,10]. Bcl-2 inhibits caspase activity and prevents the release of cytochrome c from the mitochondrion by binding to apoptotic protease activating factor-1 (APAF-1). Although the mechanism by which SURVIVIN inhibits apoptosis is not fully understood, it is known to block the apoptosis-inducing factor (AIF)-dependent apoptosis pathway; it also binds to pro-caspase-9 in association with a cofactor and selectively suppresses the mitochondria/cytochrome c apoptosis pathway. It can also bind to the effector cell death proteases caspase-3 and -7 and inhibit caspase activity and cell death. Expressed SURVIVIN associates with mitotic spindle microtubules and has oncogenic properties by overriding the G2-phase M checkpoint [11,12]. SURVIVIN expression is minimal or absent in normal adult cells, while it is high in several cancers, including melanoma [13,14]. Previous studies have also shown how inhibition of SURVIVIN expression can induce chemo-/radiosensitivity and apoptosis in melanoma cells [15,16]. We hypothesized that SURVIVIN expression may be associated with a worse clinical response in patients with in-transit melanoma metastases treated with hyperthermic antiblastic limb perfusion (ILP). Our hypothesis is that SURVIVIN gene expression levels correlate with clinical response to ILP treatment and with relapse-free (in patients with complete response to ILP), progression-free (in patients with partial response to ILP), and overall survival.

### 1.2. MDM2

p53 suppresses tumor growth and has proapoptotic (mitochondrial) activity through transcriptional activation of proapoptotic Bcl-2 family members and repression of other antiapoptotic Bcl-2 family proteins. In addition, p53 promotes receptor-mediated cell death through FAS transactivation and TRAIL-R2 transcription. The p53 tumor suppressor is therefore very important in preventing the transformation of a damaged cell into a real tumor. When a cell is exposed to “stress”, such as DNA damage, different signaling pathways are activated, resulting in different variants of p53 [17,18,19,20]. Intracellular p53 levels increase, and p53 is simultaneously activated as a transcription factor. p53 regulates stress-specific transcriptional response programs, leading to growth arrest, senescence, or apoptosis. Underlining the primary role of p53 in these processes, there is evidence that p53 is altered in about half of all cancer cases. In addition to inducing the above mechanisms, p53 also regulates its own intracellular levels through a feedback loop mechanism with MDM2. Activation of p53 induces transcription of MDM2, which binds to p53 and inactivates it, but also acts as an E3 ubiquitin ligase, causing the destruction of p53 by proteasomes [21]. The MDM2 protein regulates the intracellular levels of p53; therefore, its expression could have an oncogenic effect. In humans, expression of MDM2 and reduction/loss of p53 function is common in many types of cancer [22]. However, as previous studies have shown that p53 mutations are rare in melanoma and because both p53 “loss” and MDM2 expression are mechanisms that work by blocking p53 function in the tumor suppressor pathway, we decided to focus our attention on MDM2. There is evidence in the literature that p53 is even expressed in many tumor types (a seeming paradox) and that there is not always a correlation between p53 and MDM2 levels. This apparent paradox could be explained by the hypothesis that the presence of a mutated and functionally “inactive” p53 does not lead to an elevation in MDM2 levels and therefore is not degraded. In this case, elevated levels of “inactive” p53 and normal levels of MDM2 would be found [23]. As preclinical studies have shown that p53 is involved in the regulation of the cytotoxic effect of TNF and that MDM2 causes the destruction and inactivation of “active” p53, we hypothesized that expression of MDM2 may be one of the mechanisms that confer chemoresistance to TNF and correlate with a worse clinical response to ILP with TNF and melphalan. The aim of the study was to evaluate the correlation between treatment response and clinical outcome of patients treated with ILP for in-transit cutaneous melanoma metastases and cancer levels of SURVIVIN and MDM2.

## 2. Materials and Methods

The study was a retrospective cohort study on the influence of SURVIVIN/MDM2 expression levels on treatment response and clinical outcomes in isolated limb perfusion for in-transit cutaneous melanoma metastases.

### 2.1. Patient Group

From our historical database of all patients with in-transit metastases from malignant melanoma series, we selected a sample of 62 patients with in-transit metastases from cutaneous melanoma not amenable to surgical resection and with a previous primary melanoma with Clark levels from III to V, with T from 2 to 4, were in stage III, had undergone ILP of the limb (upper or lower) with TNF and melphalan, and had been diagnosed and/or treated for primary melanoma at the Veneto Institute of Oncology and at the University Hospital of Padua between 1998 and 2013. There were 21 males and 41 females with a mean age of 65 years, ranging from 41 to 79 years. All cases presented with bulky disease (with in-transit metastases >3 cm in diameter or with a number of lesions >15).

### 2.2. Treatment Plan

Patients, male and female, with in-transit metastases from cutaneous melanoma not amenable to surgical resection who underwent ILP of the limb (upper or lower) with TNF and melphalan in our Institute were included in the study. Patients were treated by isolated hyperthermic limb perfusion (ILP) with melphalan and TNF according to the following regime.

The major artery and vein were clamped at the desired level, collateral vessels were ligated, and a tourniquet was applied around the root of the limb proximal to the ILP region. After the catheters were inserted into the vessels (femoral or iliac for the lower limb and axillary for the upper limb), the isolated limb was perfused with extracorporeal circulation, oxygenated, and propelled by a heart–lung machine. A probe for radioguided surgery was placed at the height of the heart, and the possible leakage of the drug into the systemic circulation was checked through the administration of albumin marked with technetium in the circuit. In the absence of significant leakage, a dose of 1 mg of TNF was administered in the perfusate, and after 15 min, a dose of melphalan of 13 mg/L limb volume for the upper limb and 10 mg/L limb volume for the lower limb was administered. The ILP had a duration of 60 min from the administration of TNF. During ILP, adequate tissue temperatures were achieved and maintained by heating the heparinized perfusate and applying a heated blanket around the limb. The temperature of the limb was kept between 38.5 and 40.5 °C (mild hyperthermia). At the end of ILP, 60 min after the administration of TNF, the perfusate was drained and the limb rinsed with an electrolyte solution. The tourniquet was then released, the catheters were removed, and the vessels were sutured. An in-transit metastasis biopsy was performed for each patient in the operating room prior to performing the ILP, and the samples were stored in our tissue bank.

### 2.3. Tissue Sampling

In-transit metastasis biopsies were analyzed using the following procedures (Supplementary File S1):

- Extraction of RNA from tissue;
- Quantification and quality control of RNA;
- Retro transcription and RT-qrtPCR.

### 2.4. Response

Radiological response was assessed according to the Response Evaluation Criteria in Solid Tumors (RECIST) version 1.1. Tumor response, understood as clinical response, was assessed 2 months after ILP. The tumor response was classified as follows. Complete response (CR) was defined as clinically evident disappearance of all active tumor lesions for at least 4 weeks. A partial response (PR) was defined as a reduction of 50% or more in the total diameter of the lesion over the last month without the appearance of new lesions. When we talk about “no response”, we mean the reduction in tumor mass of less than 50% or when there was an increase of less than 25%. Disease progression was classified as occurring when there was a >50% increase in size of the tumor lesion or the appearance of new lesions, or both.

### 2.5. Survival

Patients’ clinical prognosis was assessed by analyzing disease-free survival (DFS) and overall survival (OS), i.e., the time interval between perfusion and disease recurrence or progression.

### 2.6. Toxicity

Systemic toxicity was assessed according to World Health Organization (WHO) criteria. Local toxicity was assessed according to the Wieberdink classification (Table 1).

### 2.7. Statistical Analysis

The following parameters were considered in the analysis of tumor response to ILP: characteristics of the primary tumor (Breslow thickness, Clark level, and mitotic count; ulceration was not considered as it was not available for all patients), patient characteristics (age and sex), and MDM2 and SURVIVIN levels. Univariate analysis for response was performed using Fisher’s test and logistic regression. The association of MDM2 and SURVIVIN variables with response to ILP was adjusted for patient and tumor characteristics using multivariate logistic regression. Disease-free survival (locoregional) and overall survival were calculated from the date of perfusion to local recurrence (in the case of complete responses to ILP) or disease progression (in case of partial and no-change responses) and last follow-up or patient death, respectively. For univariate analysis, a proportional hazards Cox regression model was used to calculate the hazard ratios for local progression and overall survival. The association of MDM2 and SURVIVIN variables with disease-free survival and overall survival was adjusted for patient and tumor characteristics using multivariate Cox regression. Statistical significance was set at 5% for all analyses. All analyses were performed using the Stata 12.0 software.

## 3. Results

### 3.1. Response to ILP

We found complete clinical response to ILP with melphalan and TNF in 25 patients (40% of cases), partial response in 28 patients (45% of cases), and no response/progression in 9 patients (15% of cases). In patients with MDM2 expression only (7 patients, 11% of cases), there were 2 complete responses (28% of cases), 4 partial responses (58% of cases), and no response/progression in 1 patient (14% of cases). In patients with SURVIVIN expression only (6 patients, 10% of cases), there were 3 complete responses (50% of cases), 2 partial responses (33% of cases), and no response/progression in 1 patient (17% of cases). In patients with both SURVIVIN and MDM2 expression (30 patients, 48% of cases), there were 9 complete responses (30% of cases), 16 partial responses (53% of cases), and no response/progression in 5 patients (17% of cases). In patients without SURVIVIN and MDM2 expression (19 patients, 31% of cases), there were 11 complete responses (58% of cases), 6 partial responses (32% of cases), and no response/progression in 2 patients (10% of cases).

### 3.2. Response and Survival (Descriptive Analysis)

#### 3.2.1. Group A (Expression of MDM2 Only)

In the 7 cases, there were 2 complete responses after isolated limb perfusion (ILP). Both patients relapsed, with DFS of 11 and 48 months (considering the time interval between the date of perfusion and disease recurrence or progression). Both died, with overall survival (OS) of 19 and 72 months. There were 4 partial responses, all of which progressed (DFS of 3, 6, 1, and 36 months) and resulted in death (OS of 36, 18, 19, and 42 months). One patient had no response to ILP treatment (DFS of 0 months), and he died after one year (OS of 12 months).

#### 3.2.2. Group B (Expression of SURVIVIN Only)

There were six patients in total in this group. Of these, 3 had a complete response after ILP. In one case, there was no relapse but the patient died after about 5 months, another patient was alive and disease-free with DFS and OS of 120 months, and the third patient had locoregional relapse with DFS of 58 months and OS of 84 months. There were 2 partial responses (DFS of 8 and 3 and OS of 18 and 3 months), and 1 patient had no response/progression (DSF of 0 months and OS of 9 months).

#### 3.2.3. Group C (Expression of SURVIVIN and MDM2)

There were 30 patients in this group. There were 9 complete responses. Of these, 7 relapsed, with a mean DFS of 34 months, ranging from 5 to 153 months, and with a mean OS of 54 months, ranging from 11 to 186 months. The other 2 did not relapse and died of distant metastases (DFS and OS of 43 and 93 months, respectively). There were 16 partial responses. Of these, 14 had a locoregional progression, with a mean DFS of 10 months, ranging from 1 to 24 months, and subsequently died, with a mean OS of 22 months, ranging from 7 to 40 months. Two patients showed no locoregional progression, and of these, one patient died from distant metastases (OS of 15 months), and one patient had stable disease at last checkup (OS of 48 months). In 5 patients, we found no response/progression of disease after ILP. These patients all died of the disease, with a mean OS of 24 days, ranging from 7 to 60 months.

#### 3.2.4. Group D (No Expression of SURVIVIN and MDM2)

There were 19 patients in this group. There were 11 complete responses. Of these, 3 had a locoregional relapse, with a DFS of 4, 9, and 27 months and with an OS of 9, 36, and 56 months; the other 8 did not relapse, and of these, 5 patients died from distant metastases (mean OS of 49 months, ranging from 20 to 104 months), 1 patient died from other causes (OS of 6 months), and 2 patients were disease-free at last checkup, with DFS and OS of 120 months. There were 6 partial responses. Of these, 4 had locoregional progression, with a mean DFS of 7 months, ranging from 1 to 16 months, and subsequently died, with a mean OS of 16 months, ranging from 8 to 22 months. Two patients showed no locoregional progression, and of these, 1 patient died of other causes (OS of 4 months) and 1 patient had stable disease at last checkup (OS of 64 days). In 2 patients, we found no response/progression of disease after ILP. These patients died of the disease with OS of 4 and 15 months.

### 3.3. Response and Survival (Univariate and Multivariate Analysis)

#### 3.3.1. Response to Perfusion

We considered MDM2 and SURVIVIN as categorical variables. In univariate analysis, response to ILP (Table 2A) correlated with high MDM2 (*p* = 0.039). No statistically significant differences (Table 2B) were found for SURVIVIN expression (Table 2B) (*p* = 0.426). On logistic regression (Table 2C), we found that high MDM2 was three times more likely to not respond to ILP with melphalan and TNF than low MDM2 (*p* = 0.041). The difference found for SURVIVIN was not statistically significant (*p* = 0.427). Multivariate analysis considering patient characteristics, tumor characteristics, and MDM2 levels showed that MDM2 was an independent prognostic factor (Table 2D) that was statistically significantly correlated with complete response to ILP (*p* = 0.04).

#### 3.3.2. Survival

We considered MDM2 and SURVIVIN as categorical variables. We found that high MDM2 is a factor that independently correlates (Table 3A) with local disease-free time (hazard ratio of 1.88 and 95% confidence interval of 0.965–3.669; *p* = 0.063). For SURVIVIN, we found a difference, although not statistically significant.

There was no statistically significant correlation between MDM2 levels and overall survival, nor between SURVIVIN levels and overall survival (Table 3B).

### 3.4. Complications and Toxicity

No patients had serious complications related to ILP in the postoperative period; the average drug leakage into the systemic circulation during the procedures was 3.5%, ranging from 0% to 8%. A total of 65% of patients experienced grade I or II local toxicity (according to the Wieberdink classification), with no cases of major toxicity. There were no cases of systemic toxicity.

## 4. Discussion

In this study, we observed the presence of MDM2 and SURVIVIN expression in in-transit metastases of melanoma and the correlation between this and the clinical response to ILP with TNF and melphalan. The hypothesis was that MDM2 and SURVIVIN may be involved in the chemoresistance mechanisms of TNF melanoma metastases, interfering with its cytotoxic effect and, in particular, direct toxicity mediated by apoptosis [21]. SURVIVIN is a member of the inhibitor of apoptosis protein (IAP) family and has oncogenic properties whose mechanisms are not yet fully understood [24,25]. SURVIVIN expression is minimal or absent in normal adult cells but is high in several cancers, including melanoma [21]. MDM2 regulates intracellular levels of p53, inactivating it or promoting its destruction. The oncosuppressor p53 suppresses tumor growth and has proapoptotic activity through the transcriptional activation of proapoptotic Bcl-2 family members and repression of other antiapoptotic Bcl-2 family proteins; it also promotes receptor-mediated cell death. In melanoma, p53 mutations are rare, so we decided to look at MDM2 levels [26]. In our patients, we found significant levels of SURVIVIN and MDM2 in 48% of cases, which we believe is high, and in these patients (total case series), complete responses were 40% and global responses (complete + partial) were 85%. It is interesting to note how these correspond to the response rates to ILP using melphalan alone. In patients with SURVIVIN expression only, we found approximately the same response rates (50% complete responses and 83% overall responses). In patients with MDM2 expression, alone or in combination with SURVIVIN, complete responses to ILP were significantly reduced (28% and 30%, respectively), while overall response rates remained unchanged (86% and 83%, respectively). Patients with no expression of MDM2 and SURVIVIN had the highest percentage of complete responses (58%) and global responses (90%) to ILP. Regarding the response to ILP, the results of our study showed a statistically significant correlation with MDM2 levels, and patients with MDM2 expression were three times more likely to have an incomplete response to ILP with melphalan and TNF. In multivariate analysis, among the prognostic factors considered, MDM2 was found to be independently correlated with complete response to ILP. No significant differences were found for SURVIVIN expression. Focusing on the rates of relapse or progression in patients with a (complete or partial) response to ILP, it is interesting to note that there was a significant difference between the four groups. In patients with MDM2 expression only (group A), we observed relapse or locoregional progression after ILP in 100% of cases. In patients with SURVIVIN expression only (group B), we observed relapse or locoregional progression after ILP in 60% of cases. In patients with MDM2 and SURVIVIN expression (group C), we found relapse or progression in 84% of cases. In patients without MDM2 and SURVIVIN expression (group D), we found relapse or progression in only 41% of cases. On statistical analysis, we found that expressed MDM2 was a factor that independently correlated with local disease-free time (defined as relapse in patients with a complete response to ILP or progression in patients with a partial response/no change). No significant differences were observed for SURVIVIN expression. Finally, we found no statistically significant differences in overall survival based on MDM2 or SURVIVIN levels.

## 5. Conclusions

Tumor MDM2 gene expression appears to correlate with clinical response and local disease-free survival in patients undergoing limb ILP with TNF and melphalan for in-transit melanoma metastases. As the function of MDM2 is to cause inactivation of p53, which is involved in regulating the cytotoxic action of TNF, our data support the hypothesis that the variability of clinical responses observed in patients undergoing ILP with TNF and melphalan may be due, at least in part, to mechanisms of chemoresistance to TNF involving MDM2 expression. Our study is completely original; it does not fit into any other previously experienced scenario. Although it needs to be confirmed in a larger series of patients, we suggest that MDM2 expression might be used as a predictive biomarker of tumor response to ILP, thus improving the selection of patients who can best benefit from this treatment. Moreover, as MDM2 inhibitors are already available for clinical use, we propose to test them in a clinical trial to assess whether or not their administration would improve the results of TNF-based ILP in patients with in-transit melanoma metastasis.

## Figures and Tables

**Table 1:** Wieberdink grading of limb toxicity.

Grade I	No subjective or objective evidence of reaction.
Grade II	Slight erythema and/or edema.
Grade III	Considerable erythema and/or edema with some blistering; slightly disturbed motility.
Grade IV	Extensive epidermolysis and/or obvious damage to the deep tissue causing definite functional disturbances; threat or manifestation of compartment syndrome.
Grade V	Severe reaction that may necessitate amputation.

**Table 2:** (A–D) Response to perfusion.

A. Correlation between response to ILP and MDM2						
ILP	MDM2				Total	
	<1		≥1			
Complete	14 (56%)		11 (44%)		25	
Partial + NC/Prog	11 (30%)		26 (70%)		37	
Total	25		37		62	
Pearson chi^2^ = 4.2788, *p* = 0.039						
B. Correlation between response to ILP and SURVIVIN						
ILP	SURVIVIN				Total	
	<1		≥1			
Complete	12 (48%)		13 (52%)		25	
Partial + NC/Prog	14 (38%)		23 (62%)		37	
Total	26		36		62	
Pearson chi^2^ = 0.6327, *p* = 0.426						
C. Logistic Regression MDM2 and SURVIVIN						
ILP	Odds ratio	Std. Err.	Z	P > Z	[95% CI]	
MDM2 cat.	3.008264	1.624764	2.04	0.041	1.043715	8.670621
SURVIVIN cat.	1.516484	0.795486	0.79	0.427	0.5424146	4.239787
D. Multivariate analysis for the response to ILP Comparison of MDM2, Breslow thickness, Clark level						
ILP	Odds ratio	Std. Err.	Z	P > Z	[95% CI]	
MDM2 cat.	3.186804	1.794203	2.06	0.040	1.05711	9.607059
Breslow	1.087499	0.098655	0.92	0.355	0.91035	1.299116
Clark	0.611148	0.291379	–1.03	0.302	0.24005	1.555882

**Table 3:** (A,B) Analysis of local disease-free survival (DFS) (A) and overall survival (OS) (B) according to Breslow-thickness-adjusted MDM2 level and adjusted SURVIVIN levels by COX regression.

A. DFS						
	Haz. Ratio	Std. Err.	Z	P > Z	[95% CI]	
MDM2 cat.	1.881867	0.6411763	1.86	0.063	0.9651025	3.669477
SURVIVIN cat.	1.550144	0.4916303	1.38	0.167	0.8325534	2.886235
B. OS						
	Haz. ratio	Std. Err.	Z	P > Z	[95% CI]	
MDM2 cat.	1.155603	0.3127159	0.53	0.593	0.6799336	1.964043
SURVIVIN cat.	1.101254	0.291546	0.36	0.716	0.6554513	1.850268

## Data Availability

The datasets presented in this study can be found in online repositories. The names of the repository/repositories and accession number(s) can be found here: https://zenodo.org/uploads/10194549 (accessed on 22 November 2023).

## Supplementary Materials

The following supporting information can be downloaded at: https://www.mdpi.com/article/10.3390/jpm13121657/s1, File S1: Tissue Sampling procedures.
