# Supplementary material for: Do Tumor SURVIVIN and MDM2 Expression Levels Correlate with Treatment Response and Clinical Outcome in Isolated Limb Perfusion for In-Transit Cutaneous Melanoma Metastases?
Source: J Pers Med. 2023 Nov 28;13(12):1657. doi: 10.3390/jpm13121657 (PMC10744937; doi:10.3390/jpm13121657)
Supplement: Supplementary file 1 [file jpm-13-01657-s001.zip › jpm-2691549-supplementary.pdf]

**Supplementary File S1-Tissue Sampling procedures**

**2.3.1 Extraction of RNA from tissue**

Tissue homogenization (about 50 mg) was conducted with TissueLyser (Qiagen) in TRIzol. The homogenate was shaken vigorously and incubated for 5 minutes at room temperature to allow complete dissociation of the nucleoprotein complexes. For each mL of TRIzol initially used, 240 μL of chloroform was added and mixed vigorously for 15 seconds, and the sample was incubated for 3 minutes at room temperature. The sample was then centrifuged 12,000 x g for 15 minutes at + 4 °C. The result was a polyphasic preparation in which there was an organic phase at the bottom, a white interphase containing the DNA, and a clear aqueous upper phase containing the RNA. The aqueous phase was recovered and transferred to another test tube, and 0.5 mL of isopropanol was added for each mL of TRIzol used. The mixture was incubated for one hour at room temperature to allow the RNA to precipitate, then centrifuged at 12,000 x g for 10 minutes at + 4 °C, leaving an RNA pellet at the bottom of the tube. The pellet was washed twice with 75% ethanol to remove any traces of phenol, which would compromise the subsequent retrotranscription reaction. The RNA was resuspended in RNase-free water. The purification of the extracts rich in melanin, which inhibits the quantification and retrotranscription of RNA, was carried out according to the protocol n.1 indicated by Dörrie (Journal of Immunological Methods 313 (2006) 119-128) using a column prepared in the laboratory with the Bio-Gel P-60 matrix (Bio-Rad, Hercules, CA, USA).

**2.3.2 Quantification and quality control of RNA**

To assess the quantity and quality of the RNA obtained from the extraction process, an aliquot of the extract was measured spectrophotometrically using NanoDrop (Thermo). Quantification was performed by measuring the absorbance at 260 nanometers (nm). Therefore, by carrying out the ratio between the absorbances at 260 and 280 nm, it was possible to estimate the quality of the RNA obtained (generally in pure preparations, this ratio has values between 1.9 and 2.1).

**2.3.3 Retrotranscription and RT-qrtPCR**

The RT-qrtPCR technique was used in two phases, with an initial retrotranscription reaction and subsequent qrtPCR runs. The High-Capacity cDNA Reverse Transcription Kit (Applied Biosystems), based on the use of random primers, was used for the retrotranscription. For each sample, a quantity was taken that yielded 7.00 μg of RNA in 100 uL of final reaction volume. According to the kit protocol, the reaction mix (containing water, reaction buffer, random primers, dNTPs, and MultiScribe reverse transcriptase) was added to the RNA of the samples and placed in the 9700 GeneAmp PCR System (Applied Biosystems) thermal cycler, with the following program: Stage 1: 25 °C for 10 minutes (enzyme activation); Stage 2: 37 °C for 120 minutes (retrotranscription reaction); and Stage 3: 85 °C for 15 minutes (inactivation of reverse transcriptase). The obtained cDNA was then stored at –80 °C. For the second phase of cDNA amplification, the TaqMan Gene Expression Assay protocol was followed. A 96-well plate was loaded in the following order:

- TaqMan Universal PCR Master Mix with AmpErase UNG (2X);
- Nuclease-free water;
- The TaqMan kit containing the specific primers and probe for the gene of interest.

The DNA samples of the patients were then loaded in triple technical replication. Finally, the 7300 Real-Time PCR machine software was configured.

Sample amplification conditions:

Stage 1: 50 °C for 2 minutes. The UNG enzyme was activated and carried out its action.

Stage 2: 95 °C for 10 minutes. Initial denaturation and activation of the polymerase.

Stage 3: 50 cycles, each consisting of two stages: Stage 1: 95 °C for 15 seconds (denaturation) and Stage 2: 60 °C for 1 minute (annealing and extension).

The acquisition of the signal (data collection) for each cycle was performed during Stage 2.

The GAPDH gene (glyceradehyde-3-phosphate dehydrogenase) was used as the calibrator, while AACT was used as the analysis method; the amplification threshold was calculated using the software supplied with the 7300 Applied Biosystems machine.
